# Supplementary material for: Comprehensive multimodal deep learning survival prediction enabled by a transformer architecture: A multicenter study in glioblastoma
Source: Neurooncol Adv. 2024 Jul 11;6(1):vdae122. doi: 10.1093/noajnl/vdae122 (PMC11327617; doi:10.1093/noajnl/vdae122)
Supplement: vdae122_suppl_Supplementary_Material [file vdae122_suppl_Supplementary_Material.docx]

# Self-supervised Learning Tasks

**Context Restoration**: In this procedure, a decoder module comprising transposed convolution layers, is employed to reconstruct the encoded augmented volumes. This process enables the encoder to gain an understanding of the structure, and the anatomical context of the brain regions. The optimization of the reconstruction process is achieved through the minimization of the L1 loss between the reconstructed volume and the input ground truth. Figure 1 demonstrates the reconstruction results compared to the input ground truth after training.

**Contrastive Learning**: The main goal of self-supervised contrastive learning is to learn representations that capture discriminative and useful information about the data. It does so by leveraging the concept of contrastive loss, which maximizes the similarity of the representation vectors if they are obtained from two augmented versions of the same volume, and minimizes it otherwise. The representation vector $z$ is produced by attaching a linear projection head at the output of the ViT encoder. The contrastive loss between a pair of representation vectors $z_{i}$ and $z_{j}$ is formally expressed as:

$$\mathcal{L}_{i,j}= -\log\frac{\exp\left( {\text{sim}\left( z_{i},z_{j} \right)}/\tau\right)}{\sum_{k=1}^{2N} \mathbb{I}_{\left[ k\neq i \right]}\mathrm{ex}p \left( {\text{sim}\left( z_{i},z_{k} \right)}/\tau\right)},$$

( 1 )

where $N$ is the batch size, $\tau$ is a temperature parameter, sim$\left( x,y \right)$ is the cosine similarity, and $\mathbb{I}_{\left[ k\neq i \right]}$ is an indicator function evaluating to $0$ if $k = i$ and to 1otherwise. The utilization of a contrastive learning loss function serves to enhance both the compactness within classes and the separability between representations. Given that the structure of the brain is generally similar among the population, the contrastive loss is regularized by the reconstruction loss.

# DeepHit

The loss function employed for training this model consists of two integral components. The first component, $\mathcal{L}_{1}$, serves to penalize any misalignment in the ordering of pairs concerning each specific event. Consequently, the minimization of this loss effectively promotes the accurate arrangement of pairs concerning individual events, and it is defined as:

$$\mathcal{L}_{1} = -\sum_{k=1}^{K} \alpha_{k}\cdot\sum_{i\neq j} A_{k,i,j}\cdot\text{exp}\left( -\left( \hat{F}_{k}(s^{\left( i \right)}| x^{\left( i \right)})-\hat{F}_{k}(s^{\left( i \right)}| x^{\left( j \right)}) \right)/\sigma\right),$$

where $\alpha_{k}$is chosen to trade balance between ranking losses associated with the $k^{th}$ competing event. Furthermore, $A_{k,i,j}$ is an indicator function denoting pairs $(i,j)$ that encounter risk $k$ at different time points. This function is defined as:

$$A_{k,i,j}\mathbb{\triangleq I(}\left( k^{\left( i \right)}=k, s^{\left( i \right)}<s^{\left( j \right)} \right).$$

The second component, $\mathcal{L}_{2}$, embodies the log-likelihood term, aimed at capturing the joint distribution of the first event time and events while considering the presence of right-censored data. It can be expressed as:

$$\mathcal{L}_{2} = -\sum_{i=1}^{N} \left[ \mathbb{I}\left( k^{\left( i \right)}\neq\emptyset\right)\cdot\log\left( y_{k^{\left( i \right)}, s^{\left( i \right)}}^{\left( i \right)} \right)\mathbb{+ I}\left( k^{\left( i \right)}=\emptyset\right) \cdot\log(1-\sum_{k=1}^{K} \hat{F}_{k}(s^{\left( i \right)}| x^{\left( i \right)})) \right],$$

where $\mathbb{I}\left( \cdot\right)$ is an indicator function, and $\hat{F}_{k}(s^{\left( i \right)}| x^{\left( i \right)})$ is an estimate of the Cumulative Incidence Function (CIF), which quantifies the likelihood of event $k$ happening on or before a specific time point, and it is approximated by $\sum_{m=0}^{s} y_{k,m}$. The CIF plays an integral role in deriving the survival function of each patient, which can be obtained as:

$$S(t) = 1 -\text{ }\hat{F}_{k}(s^{\left( i \right)}| x^{\left( i \right)})$$

In this work, $K$ is set to 1, as the single event of death is considered for prediction of the patient-specific overall-survival function.

Table 1 Proportional hazard test for the UCSF dataset

| Covariate | Test Statistic | P-value |
| --- | --- | --- |
| Age | 2.76 | 0.10 |
| Resection > 90% | 5.69 | 0.02 |
| Gender | 0.33 | 0.57 |
| IDH1 | 4.97 | 0.03 |
| MGMT | 0.50 | 0.48 |

Table 2 Proportional hazard test for the UPenn dataset

| Covariate | Test Statistic | P-value |
| --- | --- | --- |
| Age | 4.38 | 0.04 |
| Resection > 90% | 1.87 | 0.17 |
| Gender | 0.40 | 0.53 |
| IDH1 | 3.25 | 0.07 |
| MGMT | 0.34 | 0.56 |

Table 3 Data split for the UPenn dataset

| Variable | Train (n = 264) | Validation (n = 57) | Test (n = 57) |
| --- | --- | --- | --- |
| Age (years), mean ± SD | 63.9 ± 11.4 | 62.6 ± 13.2 | 64.3 ± 11.5 |
| Median overall survival, days | 372.5 | 351.0 | 438.0 |
| Sex |  |  |  |
| Male, n (%) | 163 (66.3%) | 32 (56.1%) | 37 (64.9%) |
| Female, n (%) | 101 (33.7%) | 25 (43.9%) | 20 (35.1%) |
| Extent of Resection |  |  |  |
| GTR [> 90% resection], n (%) | 154 (58.3%) | 31 (54.4%) | 29 (50.9%) |
| NTR [< 90% resection], n (%) | 100 (37.9%) | 24 (42.1%) | 22 (38.9%) |
| NA, n (%) | 10 (3.8%) | 2 (3.5%) | 6 (10.2%) |
| MGMT Status |  |  |  |
| Unmethylated, n (%) | 99 (37.5%) | 21 (36.8%) | 19 (33.3%) |
| Methylated, n (%) | 49 (18.6%) | 13 (22.8%) | 10 (17.5%) |
| Not available/Indeterminate, n (%) | 108 (43.9%) | 23 (40.4%) | 28 (49.2%) |

Table 4 Data split for the UCSF dataset

| Variable | Train (n = 256) | Validation (n = 55) | Test (n = 55) |
| --- | --- | --- | --- |
| Age (years), mean ± SD | 61.8 ± 12.0 | 61.3 ± 12.5 | 61.8 ± 11.7 |
| Median overall survival, days | 361.0 | 392.0 | 374.0 |
| Sex |  |  |  |
| Male, n (%) | 154 (60.2%) | 34 (61.2%) | 30 (54.5%) |
| Female, n (%) | 102 (39.8%) | 21 (38.8%) | 25 (45.5%) |
| Extent of Resection |  |  |  |
| GTR [> 90% resection], n (%) | 148 (57.8%) | 37 (67.3%) | 28 (50.9%) |
| NTR [< 90% resection], n (%) | 108 (42.2%) | 18 (32.7%) | 27 (49.1%) |
| NA, n (%) | 0 (0%) | 0 (0%) | 0 (0%) |
| MGMT Status |  |  |  |
| Unmethylated, n (%) | 99 (25.4%) | 17 (30.9%) | 14 (25.5%) |
| Methylated, n (%) | 49 (70.7%) | 36 (65.5%) | 37 (67.3%) |
| Not available/ Indeterminate, n (%) | 108 (3.9%) | 2 (3.6%) | 5 (9.1%) |


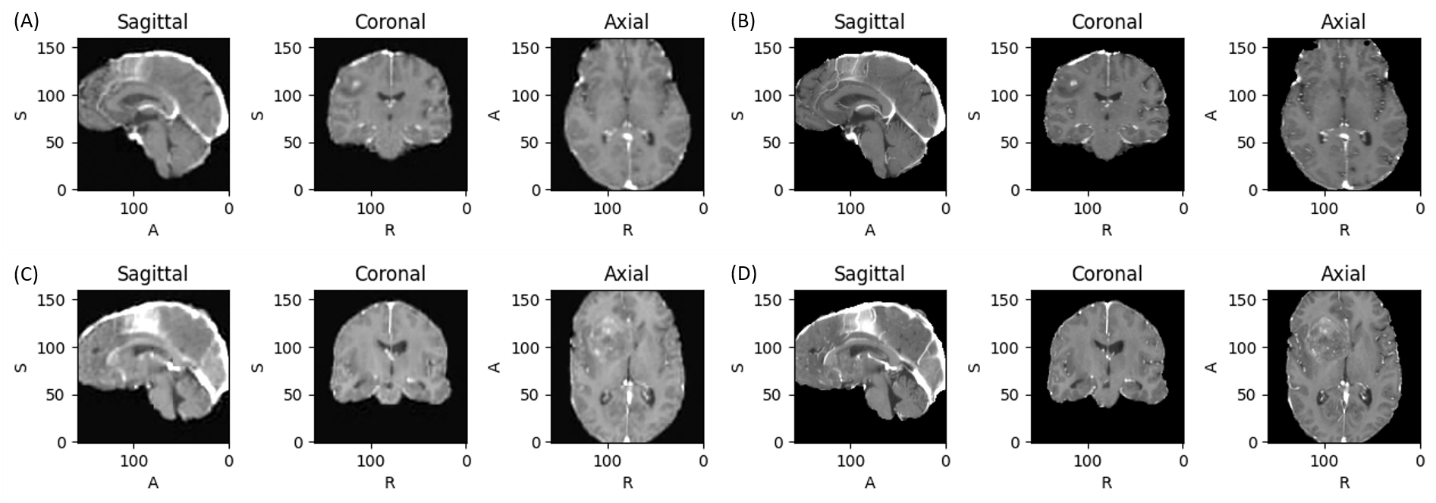


Figure 1 The reconstructed images from the trained ViT encoder-decoder (A and C) compared with the ground truth input images (B and D).
